# Series Elastic Behavior of Biarticular Muscle-Tendon Structure in a Robotic Leg

**DOI:** 10.3389/fnbot.2019.00064

**Published:** 2019-08-13

**Authors:** Felix Ruppert, Alexander Badri-Spröwitz

**Affiliations:** Dynamic Locomotion Group, Max Planck Institute for Intelligent Systems, Stuttgart, Germany

**Keywords:** robotics, bioinspired robotics, intrinsic compliance, locomotion, energy efficiency, biarticular, leg design, series elastics

## Abstract

We investigate the role of lower leg muscle-tendon structures in providing serial elastic behavior to the hip actuator. We present a leg design with physical elastic elements in leg angle and virtual leg axis direction, and its impact onto energy efficient legged locomotion. By testing and comparing two robotic lower leg spring configurations, we can provide potential explanations of the functionality of similar animal leg morphologies with lower leg muscle-tendon network structures. We investigate the effects of leg angle compliance during locomotion. In a proof of concept, we show that a leg with a gastrocnemius inspired elasticity possesses elastic components that deflect in leg angle directions. The leg design with elastic elements in leg angle direction can store hip actuator energy in the series elastic element. We then show the leg's advantages in mechanical design in a vertical drop experiment. In the drop experiments the biarticular leg requires 46% less power. During drop loading, the leg adapts its posture and stores the energy in its springs. The increased energy storing capacity in leg angle direction reduces energy requirements and cost of transport by 31% during dynamic hopping to a cost of transport of 1.2 at 0.9 kg body weight. The biarticular robot leg design has major advantages, especially compared to more traditional robot designs. Despite its high degree of under-actuation, it is easy to converge into and maintain dynamic hopping locomotion. The presented control is based on a simple-to-implement, feed-forward pattern generator. The biarticular legs lightweight design can be rapidly assembled and is largely made from elements created by rapid prototyping. At the same time it is robust, and passively withstands drops from 200% body height. The biarticular leg shows, to the best of the authors' knowledge, the lowest achieved relative cost of transport documented for all dynamically hopping and running robots of 64% of a comparable natural runner's COT.

## 1. Introduction

A persistent question in legged locomotion relates to the functional morphology of compliant elements in segmented leg structures. Elastic elements in legs enhance locomotion performance in terms of stability, robustness to perturbations and impact mitigation in legged walking systems (Hurst, 2008; Rummel et al., 2010). Leg elasticity can simplify the control task (Verstraten et al., 2016; Beckerle et al., 2017) by giving the system favorable natural dynamics. Biological observations show that muscles and tendons act like elastic elements (Biewener, 1998; Alexander, 2002) that enable rich locomotion skills with high energy efficiency at low control effort (Daley, 2008; Lakatos et al., 2018).

In bioinspired robotics, the concept of elasticity was first introduced in series elastic actuators (SEA) (Pratt and Williamson, 1995) and prismatic actuators (Raibert et al., 1984). Many robotic designs use a minimal order template, the spring-loaded inverted pendulum (SLIP) model (Blickhan, 1989; Seyfarth et al., 2001; Geyer et al., 2006), as a design baseline for walking systems. Based on SLIP models much effort has gone into designing compliance in virtual leg axis direction for robots. Compliance is implemented as either motor controlled compliance (Ding and Park, 2017; Park et al., 2017) or physical springs (Fukuoka et al., 2003; Renjewski et al., 2015; Semini et al., 2015). Like the SLIP model, these robots have elastic elements in their joints to help them achieve the same energy efficient and robust behavior as their biological role models (Alexander and Bennet-Clark, 1977).

The primary focus in designing compliance in robots using physical springs has been on *virtual leg axis direction compliance*. We pronounce the influence of additional physical elastic elements acting in *leg angle direction*. In a real world locomotion scenario we show compliance in leg angle direction to be important as well as compliance in virtual leg axis direction which has been shown in SLIP model and SLIP-inspired robots. To achieve intrinsic compliance we implement a mechanism inspired by a biological blueprint.

In studies of quadrupedal leg morphology, a four-bar-like mechanism has been observed by Lombard (1903). This simplified mechanism describes the functional morphology of lower leg muscle-tendon groups. It was extended by Witte et al. (2001, 2004) to a pantograph structure, including muscle-tendon structures. Because of the distal elastic tendon structures (Roberts, 2016), the simplified pantograph structure is spring-loaded. The concept implemented in a robot (Spröwitz et al., 2013), briefly suggested a potential function as an effective elastic element in leg angle direction (Spröwitz et al., 2014). The element is oriented so that its elastic elements possess deflection components orthogonal to virtual leg axis direction. Unlike in SLIP model, these components do not primarily contribute to deflection in virtual leg axis direction. However, they deflect under the presence of hip torque and perturbations that reflect as a torque to the hip actuator.

This leg morphology has been applied in robots before, empirically showing its advantages concerning the simplification in creating stable gaits. However, the general morphology has not yet been characterized, and the differences and advantages are not yet documented.

We investigate the effects, leg angle compliance in combination with virtual leg axis compliance has on spring behavior and resulting energy efficiency in the leg.

In this paper, we characterize one leg design with virtual leg axis compliance and one with virtual leg axis *and* leg angle compliance. We show the differences in leg morphology first on a simple kinematic model. To decompose virtual leg axis and leg angle effects we conduct static experiments to examine isolated virtual leg axis and torque influence on the elastic elements. In a drop test experiment, we investigate the mechanical behavior under dynamic loading without considering control design. At last, we compare both legs in a monoped hopping experiment and analyze the differences in dynamic behavior and energy stored and recuperated in the springs under a realistic load case.

### 1.1. Related Work

The functional morphology of multiple degrees of compliance in multi-segmented legs in animals and robotics has not been understood yet by either biologists nor roboticists. While two-segmented legs with one degree of compliance have been studied thoroughly (Raibert et al., 1984; Hutter et al., 2012; Semini et al., 2015; Park et al., 2017), the placement and interplay between multiple compliant elements is still an unsolved research topic.

Because of observations in biological examples, implementations of multi-segmented legs with several compliant elements have been tested in robotic hardware as well as in simulations to understand their behavior. Spröwitz et al. (2013, 2018) implemented a leg with a biarticular spring to investigate self stabilizing behavior on a quadruped during dynamic locomotion. They showed, that a simple sensorless central pattern generator with a position controller can allow dynamic feed forward locomotion. Iida et al. (2007) investigated the possibility to create both walking and running gaits in a humanoid biped with biarticular springs as well as the ability to create more human-like gaits. Sato et al. (2015) implemented a robot with only one biarticular spring but no intrinsic compliant knee. There, the biarticular spring provided elastic behavior to the leg for jumping and landing motions.

An aspect that has not been in the research focus yet is the interplay between both an intrinsically compliant knee and a biarticular spring in a multi-segmented leg. No systematic and comparative research exists so far comparing multiple compliant elements in highly under-actuated segmented legs, specifically for the combination of leg-angle and virtual leg axis compliance. As energy fluctuates in both directions in animal legs (Alexander, 1984) one can expect that compliant passive mechanisms evolved benefiting from these resources, i.e. energetically. We focus our research on the torque influence onto a series elastic biarticular spring and the increase in energy efficiency the additional stored energy provides.

In this paper we present a leg design with compliance in virtual leg axis direction as well as in leg angle direction. We show that the element in leg angle direction charges under torque influence, providing series elastic behavior for the hip. We show how the implementation of this element can drastically increase the amount of elastic energy stored in the leg.

## 2. Materials and Methods

### 2.1. Leg Design and Implementation

**Figure 1:**
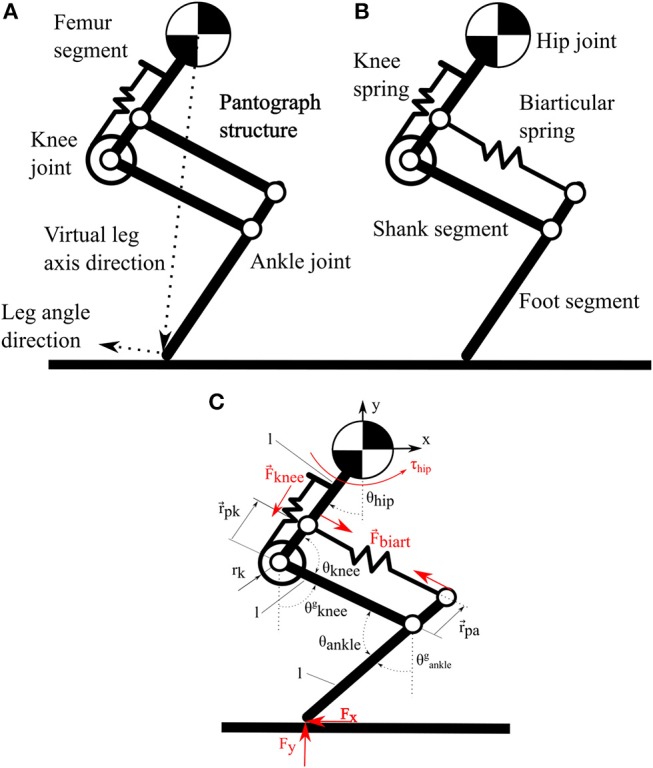
**(A)** Pantograph leg with one spring around the knee joint and a rigid pantograph segment. **(B)** Biarticular leg with one spring around the knee joint and segment with a biarticular spring. **(C)** Schematic of three segment leg with angle definitions and symbol annotations.

**Figure 2:**
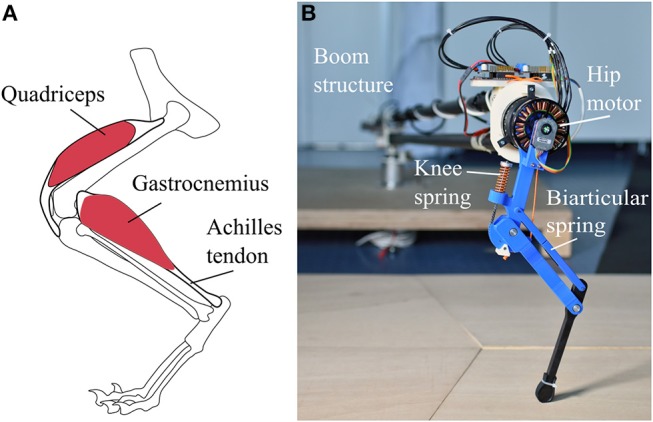
**(A)** Schematic representation of cat leg anatomy for comparison. Quadriceps originates on the upper femur and inserts into the shank segment via the patella. Gastrocnemius originates on the lower femur and inserts into the upper foot segment spanning the knee and ankle joint. **(B)** Photo of the biarticular leg mounted on the boom structure. The biarticular spring is hidden under the two parts of the biarticular segment; the point of contact is visible as a slit.

The bio-inspired leg designs under investigation (Figure 1) consist of three segments. A femur segment, a shank segment with a four-bar structure and a foot segment. The arrangement of segments and elastic elements (Figure 2B), is inspired by the leg anatomy of mammalian quadrupeds. The hip joint connects the femur segment to the trunk; the knee joint connects the hip and shank segment; the ankle joint connects the shank and foot segment. Leg segments on these legs represent the major bone groups of vertebrate animals, namely femur, tibia and fibula and the bones forming the foot segment. For simplicity, all segments in both designs have the same length.

Elastic elements in the robot are placed to mimic the functionality of big muscle-tendon groups in animal legs. In placement and functionality, the knee spring represents the quadriceps femoris muscle and patella tendon of the biological example. On the pantograph leg the segment parallel to the shank segment of the biarticular leg consists of a rigid element, forming a pantograph structure (Figure 1A). The segment parallel to the shank segment consists of a second spring connecting the hip and foot segment (Figure 1B). This biarticular segment spans two joints. The biarticular spring models the lower leg muscle-tendon apparatus of gastrocnemius muscle and Achilles tendon in a quadrupedal animal (Figure 2A). We refer to the leg with the biarticular spring as the *biarticular leg*, to the leg with the pantograph structure as the *pantograph leg*. Unloaded, the biarticular segment has the same length as the shank segment. The femur and foot segment are parallel when the biarticular spring is not deflected. Both ankle and knee joint have a hard stop to prevent over-extension.

The knee joint stiffness is realized by a spring that wraps around the knee joint on a cam mechanism, inspired by a knee cap (patella) (Allen et al., 2017; Heim et al., 2018). The knee cam mechanism linearizes the knee spring deflection over knee angle. Knee stiffness is designed to provide sufficient torque to hold the leg during running, exerting ground reaction forces three times the body weight of the robot at 10% virtual leg length deflection. The cam radius on the knee is designed to enable 35° knee angle deflection or about 70 mm leg length change. Empirically, we choose the biarticular spring stiffness similar to the knee spring stiffness, so the biarticular spring does not saturate, and the knee spring deflects similar to the pantograph leg. Through the biarticular spring the ankle joint can deflect by 60°, equivalent to 160 mm leg length change. The hip joint is articulated with a brushless motor. In combination with a 5:1 planetary gear box the nominal output hip torque is 6.2 Nm. To measure the joint deflections, all joints on the leg are instrumented with rotary absolute encoders.

The leg design consists of a hip joint rigidly connected to an actuator and two passive joints on the knee and ankle. The leg design builds on previous research on the Cheetah cub and Oncilla robots. Instead of the servo motors used in the previous robots we implemented high torque density brushless motors. To increase the backdriveability of the gear train a low ratio gearbox was used. This way the actuator can potentially be used as a proprioceptive actuator (Seok et al., 2012). The new knee spring placement in our design largely reduces the nonlinearity of the spring force to joint angle relationship of the knee joint, compared to the design used in Cheetah-cub and Oncilla. This simplifies modeling, and reduces the complexity of the mechanical design. The general mechanical leg design was improved to be more dureable and robust while at the same time reducing the complexity of the design to enable faster prototyping, as well as simplified manufacturing and assembly. The biarticular leg's lightweight design can be rapidly assembled, and is largely made from elements created by rapid prototyping. At the same time it is however robust, and passively withstands drops from 200% body height.

Here we reduce the investigation to a single leg hopping in the saggital plane. This is common practice (Semini et al., 2008; Hutter et al., 2011; Ding and Park, 2017; Liu et al., 2018). It also reduces the effects of body inertia, multiple legs and the system complexity.

### 2.2. Kinematic Model

In this section, we investigate the governing equations describing the difference in behavior for both legs. All future assertions talk about joint angles implying resultant deflection of the associated elastic elements.

By formulating the kinematic equations for the pantograph leg, we show the basics of our hypothesis. Writing the forward kinematics to obtain the foot position with the reference at the hip joint, shows that the system rank *r* = 2 with 2 parameters (θ_*hip*_ and θ_*knee*_), since θ_*hip*_ = θ_*ankle*_ because of the pantograph structure. The equation system is fully defined. In comparison, the pantograph segment in the biarticular leg is replaced by a biarticular spring. The rank of the system matrix is also *r* = 2 but because θ_*hip*_ ≠ θ_*ankle*_, an additional parameter or Degree Of Freedom (DoF) is added to the leg. Annotations are depicted in Figure 1 and Table 1.

**Table 1:** Leg parameters and robot implementation components.

**Leg Parameters**		
Segment length	*l*	150 mm
Knee and ankle resting angle		127°
Resting leg length		408 mm
Knee cam radius	*r*_*k*_	30 mm
Knee - pantograph insertion distance	*r*_*pk*_	30 mm
Mass biarticular leg		0.91 kg
Mass pantograph leg		0.88 kg
Knee spring stiffness	*k*_*k*_	10.89 $\frac{N}{mm}$
Biarticular spring stiffness	*k*_*biart*_	9.8 $\frac{N}{mm}$
**Implementation**		
Motor	TMotors MN7005 KV115	*m* = 188*g*, τ_*max*_ = 1.3*Nm*
Motor driver	TI TMS320x2806x	24 V/15 A max.
Computer	pre-empt Ubuntu 14.04	1 kHz control frequency
Joint encoders	Broadcom AEAT8800-Q24	12-bit
Planetary gearbox	Matex RS3505S	gear ratio = 1:5

*Schematic in Figure 1C*.

Forward kinematics for pantograph leg:

(1) $$\begin{array}{c} \begin{array}{ccc} x_{foot} & = & -2\cdot l\cdot \sin\left(\theta_{hip}\right)+l\cdot \sin\left(\theta_{knee}^{g}\right)\\ y_{foot} & = & -2\cdot l\cdot \cos\left(\theta_{hip}\right)-l\cdot cos\left(\theta_{knee}^{g}\right)\\ \text{for }\theta_{hip}^{g} & = & \theta_{ankle}^{g} \end{array} \end{array}$$

Forward kinematics for biarticular leg:

(2) $$\begin{array}{r} \begin{array}{ccc} x_{foot} & = & -l\cdot \sin\left(\theta_{hip}\right)+l\cdot sin\left(\theta_{knee}^{g}\right)-l\cdot sin\left(\theta_{ankle}^{g}\right)\\ y_{foot} & = & -l\cdot cos\left(\theta_{hip}\right)-l\cdot cos\left(\theta_{knee}^{g}\right)-l\cdot cos\left(\theta_{ankle}^{g}\right) \end{array} \end{array}$$

with,

(3) $$\begin{array}{r} \begin{array}{ccc} \theta_{knee}^{g} & = & \pi -\theta_{hip}-\theta_{knee}\\ \text{and}\\ \theta_{ankle}^{g} & = & \theta_{knee}^{g}-\theta_{ankle} \end{array} \end{array}$$

**Figure 3:**
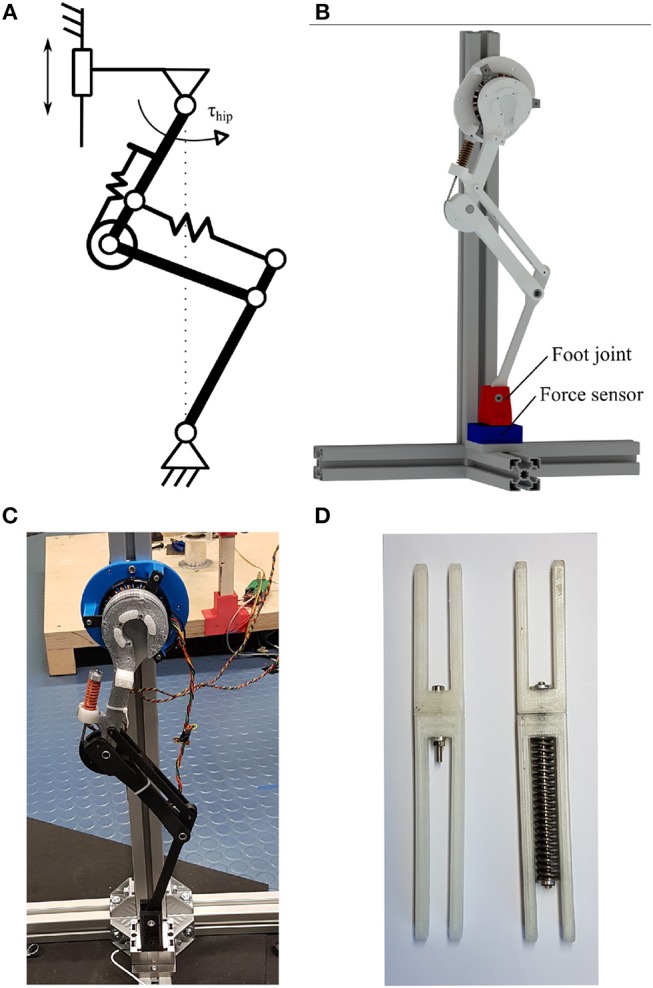
**(A)** Schematic of the static setup with the leg fixed to a rotary joint on the foot and hip joint. The fixture on the hip joint can be moved to change the virtual leg length. **(B)** Rendered leg test stand with leg fixed into a rotary joint on hip and foot (red) on force sensor (blue). **(C)** Photo of the static test setup with pantograph leg. **(D)** Photo of the two four-bar segments. Fixed pantograph segment left. Spring loaded biarticular segment right.

To describe the joint positions of the biarticular leg, an additional kinetic constraint is necessary to describe the coupling of the two springs:

(4) $$\begin{array}{r} \begin{array}{c} \frac{r_{k}\cdot F_{knee}+\vec{r_{pk}}\times \vec{F_{biart}}+\tau_{hip}}{cos\left(\theta_{hip}^{g}\right)}=\\ \frac{F_{x}\cdot l\cdot cos\left(\theta_{ankle}^{g}\right)+\vec{r_{pa}}\times \vec{F_{biart}}}{cos\left(\theta_{ankle}^{g}\right)} \end{array} \end{array}$$

(5) $$\begin{array}{r} \begin{array}{ccc} F_{knee} & = & k_{k}\cdot r_{k}\cdot \Delta \theta_{knee}\\ \vec{F_{biart}} & = & k_{p}\cdot \left(\vec{P}-l\cdot \frac{\vec{P}}{\left||\vec{P}|\right|}\right) \end{array} \end{array}$$

where, *F*_*knee*_ is the force of the knee spring and *F*_*biart*_ is the force of the biarticular spring.

(6) $$\begin{array}{r} \begin{array}{ccc} \vec{r_{pa}} & = & \left[\begin{array}{c} -||r_{pk}||\cdot sin\left(\theta_{ankle}^{g}\right)\\ \;||r_{pk}||\cdot cos\left(\theta_{ankle}^{g}\right) \end{array}\right]\\ \text{and} & \; & \;\\ \vec{P} & = & \left[\begin{array}{c} \;||r_{pk}||\cdot sin\left(\theta_{hip}\right)-l\cdot sin\left(\theta_{knee}^{g}\right)-||r_{pa}||\cdot sin\left(\theta_{ankle}^{g}\right)\\ -||r_{pk}||\cdot cos\left(\theta_{hip}\right)-l\cdot cos\left(\theta_{knee}^{g}\right)+||r_{pa}||\cdot cos\left(\theta_{ankle}^{g}\right) \end{array}\right] \end{array} \end{array}$$

where, $\vec{P}$ is the position of the biarticular spring insertion into the foot segment. If we assume the hip and foot fixed with rotary joints to a global frame (Figure 3), the pantograph leg cannot change its joint angles. Because of the increased DOF, the biarticular leg has an infinite number of joint orientations with a fixed hip and foot point.

By changing the torques and forces acting on the biarticular leg, the joint orientation can be changed based on the ratio of chosen stiffnesses. Under hip torque, the pantograph leg increases the forces on the hip and foot bearings but does not change joint angles. The biarticular leg orients its joints to satisfy the kinetic constraint described above. By changing its posture, the biarticular leg deflects the springs attached to each joint. When torque is exerted on the hip, the leg can store the energy from hip actuation in the biarticular spring. The energy storage potentially enables the biarticular leg to recuperate the energy stored in the springs.

Spring energies for leg comparison are calculated as:

(7) $$\begin{array}{r} \begin{array}{ccc} E_{knee} & = & \frac{{\left(F_{knee}\right)}^{2}}{2\cdot k_{k}}\\ \text{and} & \; & \;\\ E_{biart} & = & \frac{{\left(F_{biart}\right)}^{2}}{2\cdot k_{biart}} \end{array} \end{array}$$

where *E*_*i*_ is the energy stored in the corresponding spring, *k*_*k*_ is the knee spring stiffness, and *k*_*biart*_ is the biarticular spring stiffness.

### 2.3. Experiments

During locomotion, legs are subject to dynamic forces in leg length, as well as leg angle direction. In this section, we investigate the behavior of both legs under loads in both directions. To show the basic functionality of the leg we reduce the experiment complexity compared to a hopping experiment. In a reduced order experiment, we investigate the effects of virtual leg axis and leg angle forces separately. Then we investigate the mechanical leg behavior in a vertical drop test without control influence. Last we show that the leg shows series elastic behavior in the biarticular spring under combined loads during dynamic hopping to provide a realistic locomotion load case.

#### 2.3.1. Static Virtual Leg Axis Forces

First, we implemented a simplified setup neglecting weight and inertia effects to show the virtual leg axis related behavior clearly. Both the foot and hip joint of each leg were fixed to a ground frame by a rotational pin joint. The joint restricts both hinge points to one rotational DOF (Figure 3A). The ball bearings used to implement the rotational joints only allow forces to be transmitted, but no torques. This experiment investigates the change in joint angles purely based on change in virtual leg length. We fixed the hip joint to the frame at different virtual leg lengths in steps of 5 mm from resting leg length to 65 mm deflection, and measure the joint angles with rotary encoders.

#### 2.3.2. Static Leg Angle Torque

In the next step, we investigated the effects of hip torques on the legs in the static test stand and observed the joint angles. The legs were fixed to the same static test setup as before. Both legs were deflected by 10 mm initial leg length. We applied hip torque from 0 to 2.5 Nm in steps of 0.1 Nm every 2 s to exclude acceleration effects. We measured the resulting joint deflections as well as the forces exerted onto the foot fixture with a force sensor (K3D60 me-systeme) to verify the applied hip torque.

#### 2.3.3. Vertical Drop Experiment

**Figure 4:**
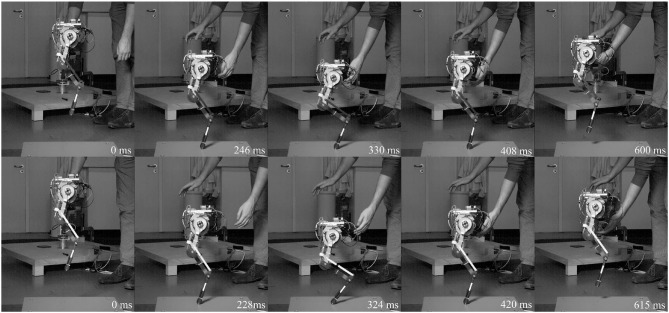
High-speed snapshots of drop experiment starting from release to next hip apex. Pantograph leg top row, biarticular leg bottom row. Depicted are left to right: drop, touchdown, maximum deflection, lift-off, apex. Due to the difference in leg stiffness the biarticular leg is on the ground longer. The biarticular spring deflection is visible in the gap in the biarticular segment at maximum deflection. The biarticular leg adapts its posture, visible in the difference of hip and foot segment angles at maximum deflection.

After investigating the static behavior of the leg, we focus on behavior under dynamic loading. We separate the effects of virtual leg axis forces and leg angle torque for vaulting the leg during forward hopping with a vertical drop experiment (Figure 4). Holding the legs at a defined position requires a motor with a position controller. We want to investigate only the mechanical response to dynamic virtual leg axis forces without effects induced by the controller. Using the same controller could give advantages to one leg, and different controllers for both legs would be hard to compare in mechanical performance. To eliminate this potential bias, we implemented a virtual spring on the hip actuator. The motor mimicked a torsion spring between the hip joint and a global frame. The virtual spring had its set point at 17.5°. At this hip angle, the virtual leg was vertical. By using a direct drive motor as a proprioceptive actuator, we avoided measuring inaccuracies through backlash, friction and reflected gearbox inertia. We used the proprioceptive actuator as a sensor to directly measure the hip angle deflection as well as the resulting forces. The virtual spring stiffness was chosen at 5.8 $\frac{Nm}{rad}$ to match the position controller gains used for the hopping experiments. The leg with joint encoders was connected to a boom structure, to restrict the motion to the sagittal plane (Figure 2B). The boom was instrumented with rotational encoders (AMT 102-V) to measure the horizontal and vertical angle of the boom representing the center of mass position of the robot. The biarticular and pantograph leg were dropped from 590 mm hip height. We also measured the input power consumption of the motor driver with a current sensor (ACS713). Data was normalized from hip dropping height to first apex (drop cycle) and averaged over 30 drops and displayed with a 95% confidence interval. Touchdown and liftoff are determined by when the spring-loaded ankle joint starts to deflect. Hip torque is calculated from armature motor current as:

(8) $$\begin{array}{r} \tau_{hip}=i_{armature}\cdot k_{t} \end{array}$$

where *i*_*armature*_, is the armature motor current measured on the motor driver and *k*_*t*_ is the torque constant of the motor. Electrical system input power is calculated as:

(9) $$\begin{array}{r} P_{el}=U\cdot I \end{array}$$

where *U*, is the constant power supply voltage of 24 V, and I is the input current measured from the power supply.

#### 2.3.4. Hopping Experiment

In this section, we provide a realistic locomotion showcase to investigate the behavior of a single hopping leg under a combination of virtual leg axis forces and hip torque. The leg is again constrained to movement in the sagittal plane without trunk rotation by a boom structure.

Both legs use the same gearbox in this experiment to provide enough hip torque for forward locomotion. We implemented a sine wave position controller on the hip actuation resulting in a hopping gait,

(10) $$\begin{array}{r} \theta_{hip_{desired}}=\theta_{0}+\theta_{1}\cdot sin\left(2\cdot \pi \cdot f\cdot t\right) \end{array}$$

where θ_*hi*_*p*__*desired*__ is the desired hip angle, θ_0_ is the hip angle offset, θ_1_ is the hip angle amplitude, *f* is the hopping frequency, and t is time.

A PD position controller calculates the desired current for the low level current controller on the motor driver according to:

(11) $$\begin{array}{rcl} i_{motor_{desired}}\left(t\right) & = & kp\cdot \left(\theta_{hip}{\left(t\right)}_{desired}-\theta_{hip_{encoder}}\left(t\right)\right)\\ \; & \; & \;+kd\cdot \frac{d\left(\theta_{hip}\left(t\right)-\theta_{hip_{encoder}}\left(t\right)\right)}{dt} \end{array}$$

where *kp* and *kd* are the proportional and derivative control gains, θ_*hi*_*p*__*encoder*__ is the measured hip angle and θ_*hi*_*p*__*desired*__ is the desired hip angle calculated above.

**Figure 5:**
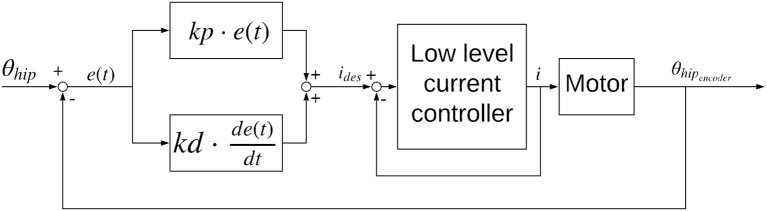
Controller diagram for the sine wave position controller used for the forward hopping experiments.

**Figure 6:**
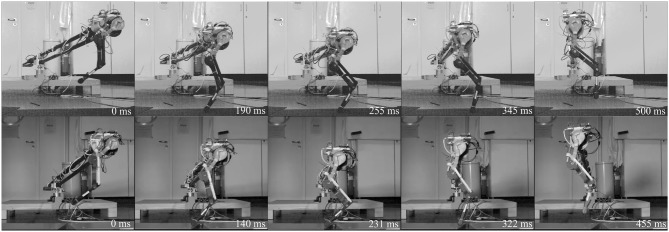
High-speed snapshots of both legs hopping forward for one step cycle from release to hip apex. Pantograph leg top row, biarticular leg bottom row. Depicted are left to right: hip apex, touchdown, maximum deflection, lift-off, second hip apex. The biarticular spring deflection is visible in the gap in the biarticular segment at maximum deflection. Timing differences stem from different duty factor and difference in gait frequency, 2 Hz for the pantograph leg and 2.2 Hz for the biarticular leg. The biarticular leg adapts its posture, visible in the difference of hip and foot segment angles at maximum deflection.

The PD position controller, schematic in Figure 5, of the motor driver was tuned to the same gains for both legs. Gait parameters θ_0_, θ_1_ and the gait frequency were hand-tuned for both legs to get stable hopping at 0.99 $\frac{m}{s}$ with *f* = 2*Hz* for the pantograph leg and 0.95 $\frac{m}{s}$ with *f* = 2.2*Hz* on the biarticular leg (Figure 6). We defined stable hopping, if the robot hopped for trials longer than 2 min, equivalent to ≅ 240 steps. Data was collected 1 min after the robot achieved a stable gait. All data sets were normalized over time from hip apex to apex (step cycle) and averaged over 30 consecutive steps. Average data was displayed with 95% confidence intervals. Touchdown and liftoff were determined by when the springs started to deflect. All future discussions are conducted using the averaged data set to get a representative picture.

## 3. Results

In this section, we present data and results from the static leg force experiment, the static leg angle torque experiment, the vertical drop test, and the hopping experiment.

### 3.1. Static Virtual Leg Axis Forces

**Figure 7:**
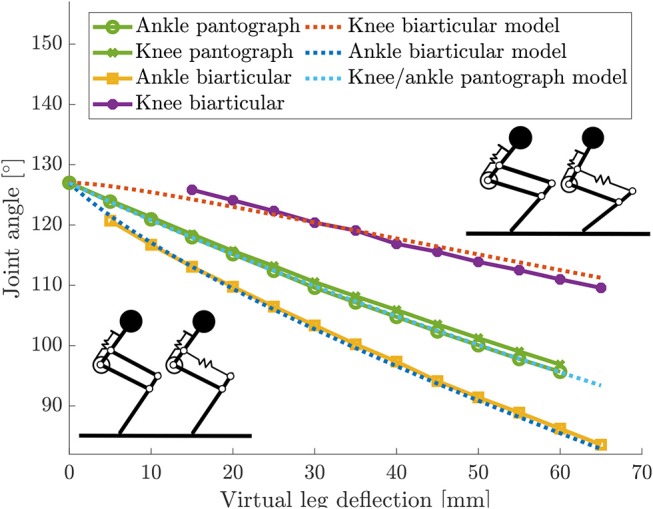
Knee and ankle angle changes from resting angles for the static virtual leg axis experiment with both legs. Knee and ankle angles change equally because of the pantograph structure. Knee and ankle change not equally in the biarticular leg because of the additional degree of freedom.

In the pantograph leg knee and ankle angles change equally (Figure 7). Because of the parallelogram geometry in the leg's four-bar mechanism, knee and ankle angles are kinematically coupled to be equal. Play in the joints causes the small deviation between the pantograph knee and ankle angle curve. At maximum leg deflection, the pantographs knee and ankle angle deflect by 32°. The model prediction fits the data, neglecting the small deviation of ≤ 2°.

In the biarticular leg, the change in knee and ankle angles are not equal. Because of the biarticular spring, the ankle deflects more than the knee. At maximum deflection the ankle in the biarticular leg deflects by 43°, the knee deflects to 17°. This first experiment shows, that knee and ankle are not kinematically coupled in the biarticular leg.

### 3.2. Static Leg Angle Torque

**Figure 8:**
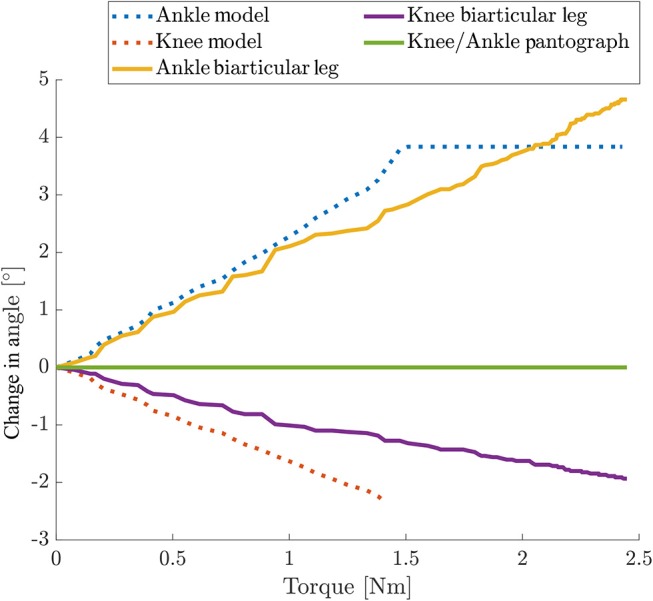
Change in joint angles over hip torque for static torque experiments for both legs. Hip position was fixed to 10 mm leg length deflection. Cut-off in the model are due to the hard stop to prevent over-extension. The cut in the experimental data is only visible as a change in slope due to material elasticity. The knee model is only valid until hitting the hard stop. Only the biarticular leg can deflect its joints under torque. Because of the kinematic coupling in the biarticular leg the knee joint deflects as well when the ankle joint deflects. In the pantograph leg the hip and foot position are fixed and the joints do not deflect under hip torque.

Because of the kinetic coupling, both knee and ankle angles in the biarticular leg change under torque (Figure 8). The knee on the biarticular leg deflects by 0.85°, the ankle deflects by 2.1° at 1 Nm. The model shows a reasonable prediction for the angles. The deviation and flat line stem from the hard stop at the knee preventing the knee from over-extension over its resting angle. The hard stop effect is less pronounced in the experimental data, due to material elasticity. It can be seen as a change in slope. The knee model is only valid before hitting the hard stop.

This experiment shows that the biarticular leg can store energy from hip actuation in the biarticular spring. Under the influence of hip torque, the pantograph leg does not change its joint angles.

We argue that the distal elastic element, mimicking the lower leg muscle-tendon structures, acts to the hip actuation like a serial spring. Different from a classical SEA hip actuator, the biarticular spring has components acting in both virtual leg axis and leg angle direction. The ratio of components that act in virtual leg axis or leg angle direction depends on the virtual leg deflection as well as the resting joint angles, and the chosen spring stiffnesses.

These experiments are abstracted from the behavior of a hopping robot. Under only hip torque the data shows that the biarticular leg has the ability to store hip actuator energy in its springs. For any given initial posture the leg can adapt its posture and store energy in the springs that can potentially be recuperated.

### 3.3. Vertical Drop Test

**Figure 9:**
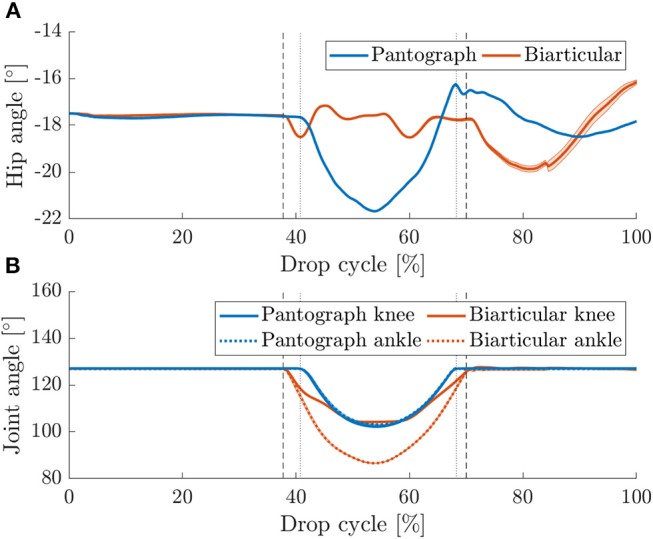
**(A)** Mean hip angles for vertical drop tests with 95% confidence levels for 30 drops. Vertical touchdown (left markers) and lift off (right markers) are plotted for pantograph (dotted) and biarticular leg (dashed). The hip angle in the pantograph leg deflects more since the leg only has one DOF. In the biarticular leg, the leg can adapt its internal posture to mitigate the dynamic forces without reflecting them to the femur segment. **(B)** Knee and ankle angles for vertical drop tests with 95% confidence levels for 30 consecutive steps. Touchdown (left markers) and lift off (right markers) are plotted for pantograph (dotted) and biarticular leg (dashed). In the pantograph leg, the joints deflect symmetrically because of the kinematic coupling. In the biarticular leg, the ankle joint deflects nearly twice as much as the knee joint.

**Figure 10:**
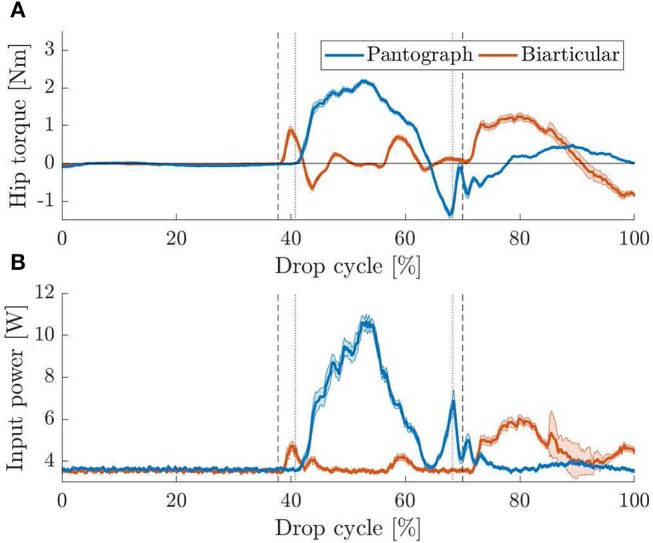
**(A)** Mean hip torque for vertical drop tests with 95% confidence levels. Vertical touchdown (left markers) and lift off (right markers) are plotted for pantograph (dotted) and biarticular leg (dashed). Because the biarticular leg adapts its posture forces are reflected less into the femur segment. This results in a lower hip angle change. **(B)** Mean input power consumption for vertical drop tests with 95% confidence levels. Touchdown (left markers) and lift off (right markers) are plotted for pantograph (dotted) and biarticular leg (dashed). In the biarticular leg, the motor does not need additional power to counteract the force. The biarticular leg requires 81% less motor power to hold the virtual leg vertical.

During stance phase (Figure 4), the hip angle in the pantograph leg deflects by 4° (Figure 9A). Because of the kinematic coupling in the leg, any force reflects into the femur segment and change its angle. Because the biarticular leg has one more DOF, it adepts its posture (Figure 9B). By changing the ankle and knee angle the energy is stored in the springs, and the data shows that the hip angle does not change. As the virtual spring induces a torque when the hip angle deflects from its resting angle, the hip torque during stance phase is much higher in the pantograph leg (Figure 10). Hip torque is calculated from measured armature current on the motor. The hip torque on the pantograph leg peaks at 2.2 Nm while the biarticular leg peaks at 1.2 Nm. Mean hip torque during stance phase is 0.22 and 0.02 Nm for the pantograph leg and biarticular leg, respectively.

After liftoff, the torque requirement is higher in the biarticular leg. Since the biarticular leg has elastic components in leg angle direction, a force resulting from unloading the joint rapidly reflects into leg angle direction. The virtual leg shoots forward when the two parts of the biarticular spring mount collide due to the hard stop. The collision can also be seen in the provided high-speed video in the Supplementary Material. We ignore this reflection effect and do not compensate or utilize it here.

The duration of stance phase varies between 27% on the pantograph leg and 32% on the biarticular leg. We suspect the difference is due to the higher mass of 29 g as well as the lower global leg stiffness of the biarticular leg.

Because the drop experiment is not a periodic motion, the beginning and end points of the graphs do not match as the leg moves differently for the subsequent lower hops.

As a result of the higher torque requirement, input power shows that the biarticular leg needs less power during stance phase to keep the desired leg posture (Figure 10B). After liftoff, the same rise in power that was explained in the hip torque curve is visible. Since oppressing the reflection effect requires high torque at high speed, a drastic rise in power consumption during swing phase is visible. Over the full step cycle, mean power consumption for the pantograph leg is 4.6 and 3.9 W for the biarticular leg. Mean power consumption during stance phase for the pantograph leg is 6.8 W for the pantograph leg and 3.7 W for the biarticular leg. The biarticular leg shows a 46% lower power requirement during stance phase and 15% lower power consumption over the whole drop cycle.

### 3.4. Hopping Experiment

**Figure 11:**
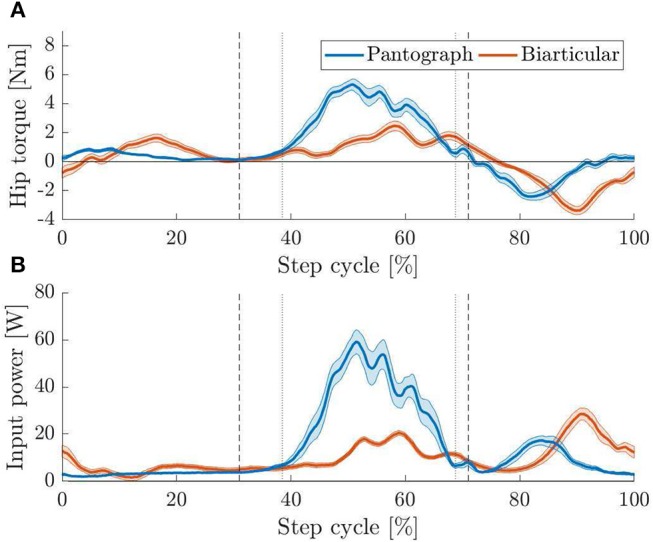
**(A)** Apex to apex normalized hip torque for both hopping legs with 95% confidence levels. Vertical touchdown (left markers) and lift off (right markers) are plotted for pantograph (dotted) and biarticular leg (dashed). During stance phase, the biarticular leg has lower peak torque requirements than the pantograph leg. After toe-off, the hip actuator suppresses the reflection effect shooting the leg forwards. The active suppression increases the torque requirements on the biarticular leg. **(B)** Apex to apex normalized input power for both hopping legs with 95% confidence levels. Touchdown (left markers) and lift off (right markers) are plotted for pantograph (dotted) and biarticular leg (dashed). Peak input power for the biarticular leg is 50% lower and mean power is 31% lower than on the pantograph leg.

During forward hopping (Figure 6) the pantograph and biarticular leg show a similar trend in torque requirements as during the drop experiments before. Hip torque is calculated from measured armature current on the motor. The peak hip torque is 5.2 Nm for the pantograph leg and 2.4 Nm for the biarticular leg (Figure 11A). The mean torque for the pantograph leg is 0.19 and 0.05 Nm for the biarticular leg over the whole step cycle. Mean torque during stance phase is 0.64 and 0.23 Nm for pantograph and biarticular leg respectively. The biarticular leg requires 53% less peak torque and 74% less mean torque.

**Figure 12:**
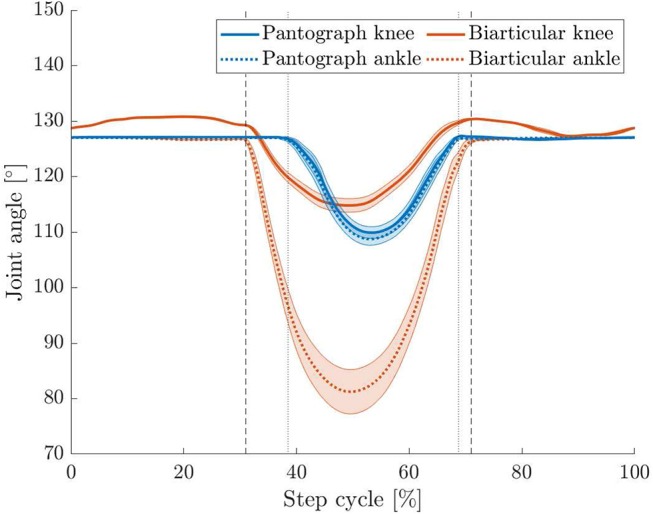
Apex to apex normalized knee and ankle angle change for both hopping legs with 95% confidence levels. Vertical touchdown (left markers) and lift off (right markers) are plotted for pantograph (dotted) and biarticular leg (dashed). The ankle angle in the biarticular leg deflects by 45° compared to 19° on the pantograph leg. Knee angles for both legs deflect similarly.

The previously observed difference in knee and ankle angle between the two legs is also visible during hopping (Figure 12). While the pantograph leg deflects both joints the same way due to the four-bar geometry, the ankle deflects more in the biarticular leg. We observe that the knee joints deflect similar in both legs. The biarticular leg's ankle, however, deflects by 45° compared to 19° on the pantograph leg.

Additionally, the duty factor, the fraction of stance phase over one step cycle period:

(12) $$\begin{array}{r} d_{duty}=\frac{t_{Stance}}{T_{Stepcycle}} \end{array}$$

is much smaller at 31% of step cycle on the pantograph leg than on the biarticular leg where the duty factor is 40%. We assume the duty factor to be higher due to the lower global leg stiffness resulting in an extended stance phase duration as the leg deflects more.

Power requirements during hopping are higher in the pantograph leg than in the biarticular leg (Figure 11B). The pantograph leg power peaks at 60 W where the biarticular leg peaks at 20 W during stance and 30 W during swing because of the reflection effect. Mean input power for the pantograph leg is 14.1 and 9.7 W for the biarticular leg. Mean power requirement on the biarticular leg is 31% lower and peak power requirement is 50% lower. The difference in input power requirement is evident in the cost of transport (COT) (Tucker, 1975),

(13) $$\begin{array}{r} COT=\frac{P_{in}}{m\cdot g\cdot v}\;, \end{array}$$

where *P*_*in*_ is electrical input power to the motor driver, m is the robot mass, g is the gravitational acceleration, and v is the forward speed of the robot.

Total COT is calculated using overall input power. The total COT for the pantograph leg is 1.7 compared to the biarticular leg at 1.2. COT when substracting 3 W idle power consumption of the system is 1.3 for the pantograph leg and 0.8 for the biarticular leg. To investigate this further, we calculate the energy stored in the springs and compare the two leg designs.

**Figure 13:**
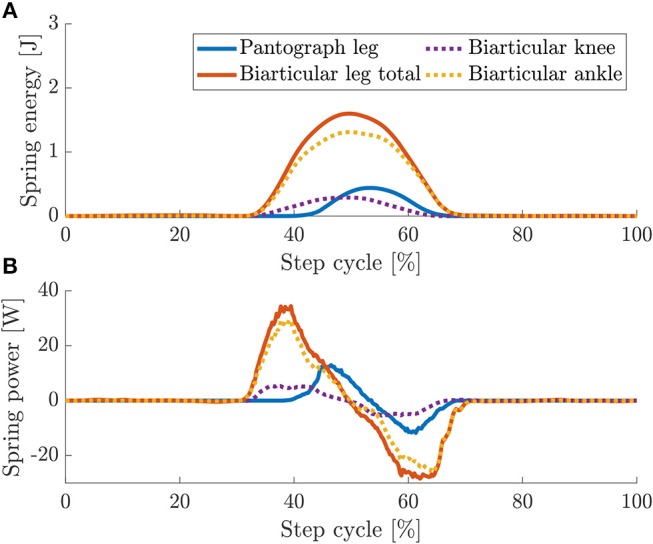
Overlaid spring energy **(A)** and power **(B)** for pantograph (orange) and biarticular leg (blue). Due to the biarticular spring, the energy stored in the biarticular leg is 2.4 times higher. In dashed lines are shown the energy and power for the individual springs in the biarticular leg. The energy in the knee springs are similar. The energy in the biarticular spring is ≅ 80% higher.

In the biarticular leg, the overall stored energy is considerably higher than the energy stored in the pantograph leg (Figure 13A). The maximum total spring energy in the pantograph leg is 0.45 J vs. 1.56 J for the biarticular leg. Mean spring energy is 0.06 and 0.34 J for the biarticular leg. Total spring energy for the pantograph leg is 82% lower than for the biarticular leg. As we show a higher energy efficiency in the biarticular leg, we conclude that the leg design has a higher recuperation rate. Higher recuperation means the biarticular leg can use the energy stored in its spring more effectively for locomotion.

To get a clearer picture on the biarticular leg, we also plot the individual spring energy contribution to the total energy. The knee spring on the biarticular leg stores roughly the same amount of energy as the single knee spring in the pantograph leg. Peak knee spring energy for the biarticular leg is 0.28 J and peak biarticular spring energy is 1.3 J. Mean energy stored in the biarticular knee is 0.05 J and mean biarticular spring energy is 0.28 J. The mean energy stored in the biarticular spring is 82% higher.

The maximum power in the springs in the pantograph leg at 12.9 W is 62% lower than in the biarticular leg at 34.4 W (Figure 13B). The maximum released power is –11.7 W in the pantograph leg and –28.4 W in the biarticular leg.

The biarticular leg stores more energy in its springs due to the elasticity in both virtual leg axis and leg angle direction. In the vertical drop and the dynamic hopping experiment, the leg recuperates more energy from its springs which reduces the overall required torque and input power.

## 4. Discussion

The placement and functional morphology of elastic elements in legs is an important research question in legged locomotion. In this paper, we show that the biarticular spring, which mimics the elasticity of lower leg muscle tendon structures, has elastic components that can provide series elastic behavior to hip actuation. In a model as well as a static experiment, we show *how* the biarticular spring enables the leg to deflect its joints at a fixed leg length without changing the hip and foot position. We then show that the additional degree of freedom allows the leg to store energy provided by hip actuation in this elastic element. In a vertical drop test with a virtual spring on the hip, we show that the favorable lower peak torque and power consumption of series elastic behavior do not depend on the motor controller but result from leg mechanics. In the drop experiment, we show that the leg changes its internal posture to adapt to external forces instead of reflecting these forces into the hip actuation. As the hip actuation does not need to compensate for the dynamic loading, no additional torque and power is required, which increases energy efficiency.

Last we show that in a combined load case of torque and virtual leg axis forces, the peak torque and power requirements are lower for the leg with distal series elastic components. By reducing the overall leg stiffness, the leg has a smaller leg length which acts as the lever arm for hip torque to produce a ground reaction force. The higher leg length deflection of 64 mm on the biarticular leg vs. 37 mm on the pantograph leg reduces the mean torque requirement for the leg.

Compared to the vertical drop the biarticular ankle joint during hopping deflects more by 6°, even though the hopping height, 490 mm for the pantograph leg and 470 mm for the biarticular leg, is lower than the drop height for the vertical drop. The difference in hip angle stems from the deflection under the additional hip torque to move the leg forward. The higher joint deflection is the result of the combined load case of virtual leg axis forces through dynamic loads and the torque required to vault the leg forward. As expected the biarticular leg stores energy provided by hip actuation in the biarticular spring even under a combined load case of virtual leg axis forces and leg angle torques.

Through the implementation of this elasticity, it is possible to reduce the peak power requirement by 26%, the mean power requirement by 31%, the peak torque requirement by 53% and mean torque by 71% in the hopping experiment.

We show that the biarticular leg with elastic components in leg angle direction possesses the same effects as a series elastic element, namely reduced torque and power requirements. We can, therefore, conclude, that the biarticular leg adds series elastic behavior to the leg. Because the biarticular spring stores 82% more energy we can further conclude that the biarticular spring also reduces the mean power and torque requirements of the leg. The reduced energy requirement shows that in robotic legs leg, compliance in leg angle direction is an equally important design parameter to virtual leg axis compliance.

**Figure 14:**
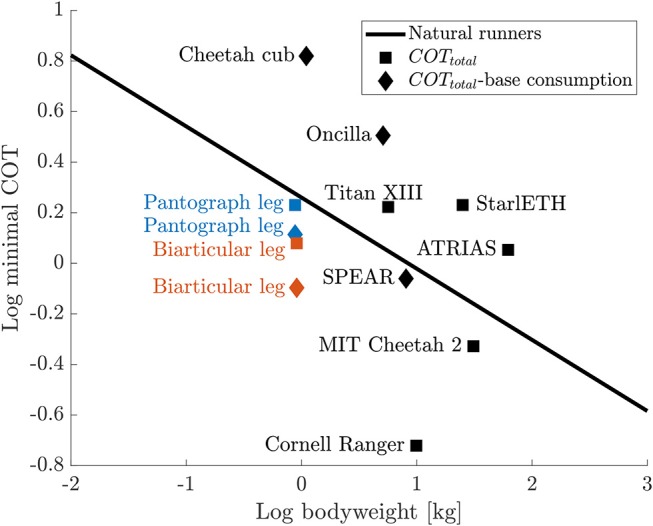
COT comparison for a selection of legged robots (Spröwitz et al., 2013, 2018; Bhounsule et al., 2014; Hutter et al., 2014; Renjewski et al., 2015; Kitano et al., 2016; Park et al., 2017; Liu et al., 2018) compared to the regression for animal runners from Tucker (1975) that shows a linear regression for the minimal COT from various running animals. Total COT values are shown as squares, COT values where base consumption (communication, electronics, etc.) is substracted, are shown as diamonds. The COT for the biarticular leg is 64% of the COT of a natural runner with the same weight. All COT values are listed in the Supplementary Material.

To put the COT of our design into perspective, Figure 14 shows the COT values for a selection of robots as well as the regression from Tucker (1975) for animal data over their respective masses. Both the pantograph leg as well as the biarticular leg are below the line for comparable natural runners. We include SPEAR (Liu et al., 2018) as a direct comparison to our monoped hopper. Comparing the COT without base consumption of our biarticular design to SPEAR, the COT of our design is lower at 0.8 than SPEAR at 0.86.

Since power, speed and mass do not scale linearly, as shown by Tucker, we believe that a better comparison than absolute COT numbers, is the comparison to a natural runner of comparable weight, the relative COT. We calculate the relative distance of the biarticular leg's COT to the COT of a model animal of the same weight from the Tucker linear regression.

Comparing the biarticular leg's total COT (including base consumption) to natural runners, the biarticular leg is still below the natural runners line and roughly on the same level as the pantograph leg without base consumption. The relative COT for the biarticular leg is 64% of a natural runner's COT. The relative COT without base consumption is at 43% of the comparable natural runner's COT. To the best of the authors' knowledge, the biarticular leg shows the lowest achieved relative cost of transport documented for all dynamically hopping and running robots including MIT Cheetah at 68% relative COT.

The only legged robots with a lower relative COT are Cornell Ranger (Bhounsule et al., 2014) and Cargo (Guenther and Iida, 2017). Cornell Rangers COT of 0.19 is 20% of the COT of a comparable natural runner. Cornell Ranger was optimized for COT efficient walking, unlike the here shown dynamic hopping locomotion of the biarticular leg. Cargos COT of 0.1 is 21% of a comparable natural runner. Cargo was designed to run at its natural frequency to increase COT. We exclude Cargo because of its non-practical ground clearance of (Guenther and Iida, 2017, Figure 12).

With the results of this paper we create a novel, robotic perspective on the placement and functional morphology of elastic elements in legs. Our research raises the question whether a transfer from the insights from this abstracted model back to biology is possible which has not been shown or discussed in previous research in biology. By showing the same joint deflection behavior under similar load cases, it might be possible, to verify the behavior of the biarticular leg in its natural role models. By finding similar behavior we could then conclude that the anatomy of vertebrate animals is in parts due to the functional morphology shown in this paper. During experimentation, we show a reflection effect that shoots the leg forward at the end of stance phase. While not considering the effect in this study, we will focus our future research on implementing controllers that utilize the effect to further reduce power requirements during the swing phase.

Additionally, we will investigate whether the distal series elastic element increases robustness to perturbations. To follow up the findings in this paper we want to optimize the energy recuperation through an investigation into the effects of posture, segmentation and spring stiffness ratio on the elastic behavior of the leg.

## 5. Conclusion

In this paper, we investigate the effects of a distal biarticular elastic element. We show that a bio-inspired distal elastic element has components that deflect in leg angle direction. To characterize the leg we provide a mathematical model, to show the underlying behavior. We then investigate the leg behavior first under virtual leg axis forces. We show that the distal elastic element provides an additional degree of freedom to the leg. In a second step we investigate the leg behavior under only leg angle torque. The second experiment shows that the elastic components in leg angle direction deflect under hip torque and store hip actuator energy. Then we show that the leg can reconfigure its internal posture during a vertical drop experiment. The leg adapts its posture to the loading force, leading to a lower femur deflection. This decreases the power requirement during drop experiments by 46% compared to the leg with only virtual leg axis compliance. The leg angle actuator will therefore require less torque and power to hold the leg during stance. Last we show that the effects investigated in reduced complexity experiments are visible in a realistic monoped hopping experiment with combined leg angle torques and virtual leg axis forces. In the hopping experiment we show that the distal elastic element reduces the power requirements by 31% and the peak torque requirements by 71%. We record a 31% reduced COT of 1.2 for our leg design of 0.9 kg at 1 $\frac{m}{s}$. The relative COT of our biarticular leg design is 64% of a comparable natural runner's COT.

## Data Availability

The datasets generated for this study are available on request to the corresponding author.

## Author Contributions

FR contributed to the concept, robot design, experimental setup, experimentation, data discussion, and writing. AB-S contributed to the concept, data discussion, and writing.

### Conflict of Interest Statement

The authors declare that the research was conducted in the absence of any commercial or financial relationships that could be construed as a potential conflict of interest.
