# Supplementary material for: Series Elastic Behavior of Biarticular Muscle-Tendon Structure in a Robotic Leg
Source: Front Neurorobot. 2019 Aug 13;13:64. doi: 10.3389/fnbot.2019.00064 (PMC6700334; doi:10.3389/fnbot.2019.00064)
Supplement: Supplementary file 1 [file Data_Sheet_1.PDF]

Supplementary Material

1 SUPPLEMENTARY TABLES AND FIGURES

1.1 Figures

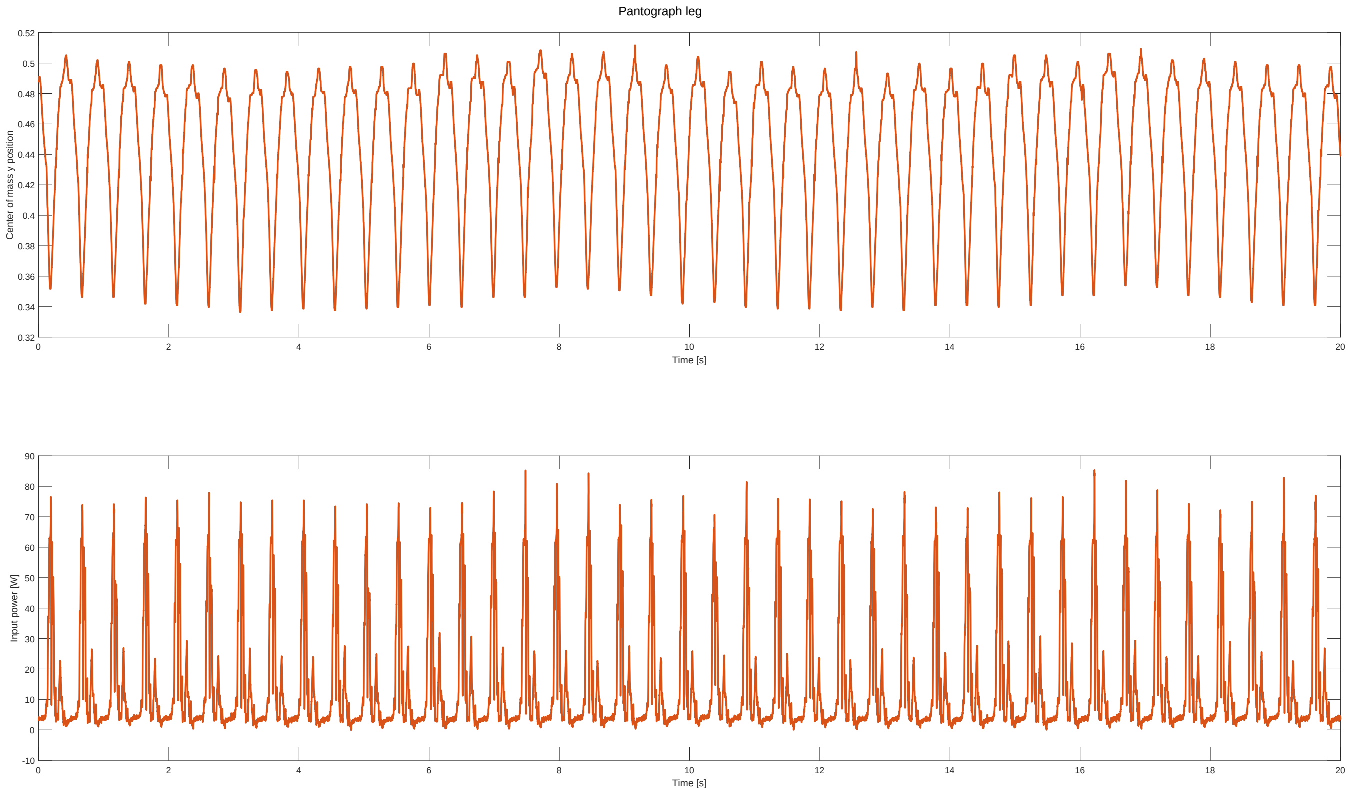

Figure S1. Raw data for pantograph leg hopping for 20 seconds of consecutive hopping. Top figure shows center of mass y position. Bottom figure shows input power measurement.

1.2 Tables

**Robot**	**Weight** [kg]	**COT** [$\frac{J}{Nm}$]	**Relative COT** [%]
MIT Cheetah	31	0.47	68
Cheetah-cub	1.1	6.6	373
Oncilla	3.2	5.1	278
Atrias	62	1.13	198
StarlETH	23	2.5	332
SPEAR	8.07	0.86	85 (-base)
Cornell Ranger	9.91	0.19	20
TITAN XIII	5.65	1.67	149
Pantograph leg	0.88	1.7 (total), 1.3 (-base)	90 (total), 69 (-base)
Biarticular leg	0.909	1.2 (total), 0.8 (-base)	64 (total), 43 (-base)

Table S1. COT and weight values for the robots compared in Fig. 14

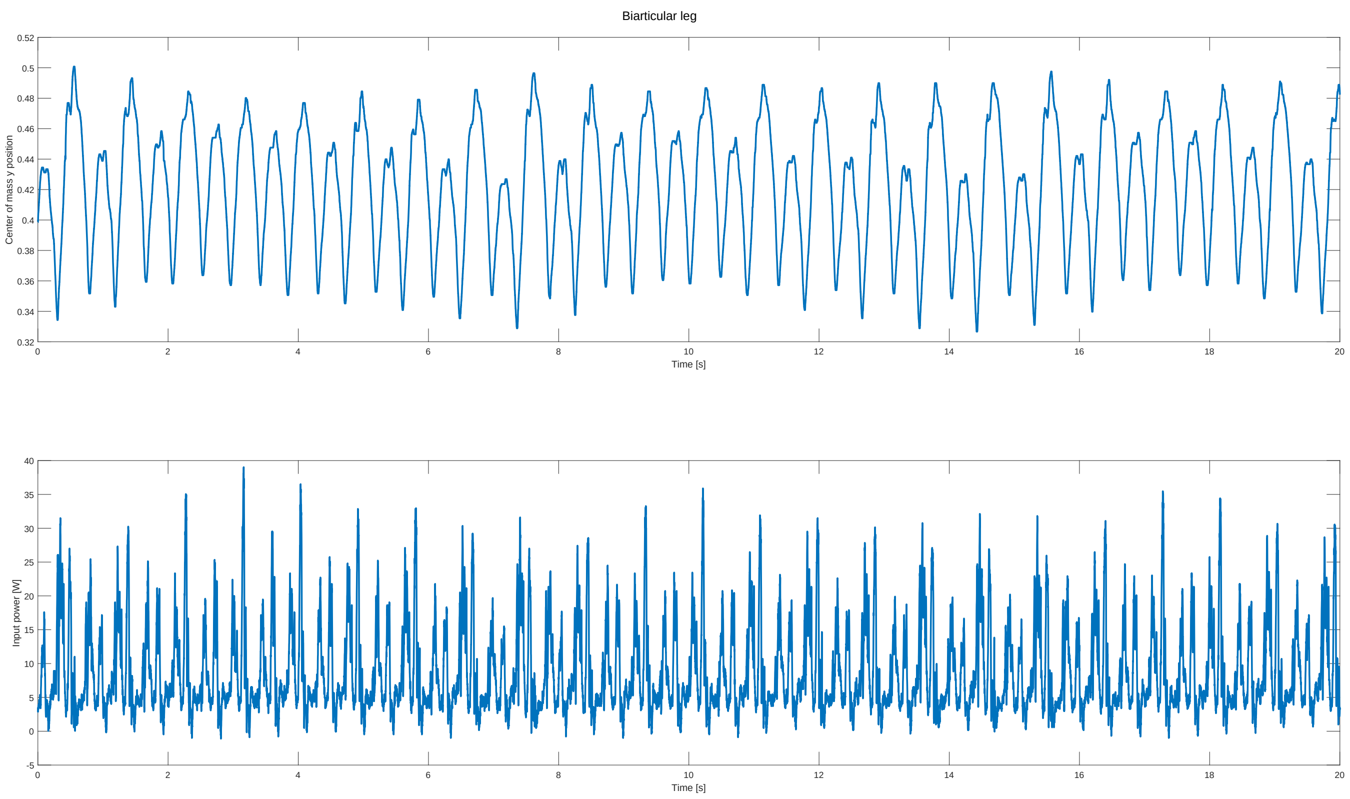

Figure S2. Raw data for biarticular leg hopping for 20 seconds of consecutive hopping. Top figure shows center of mass y position. Bottom figure shows input power measurement.

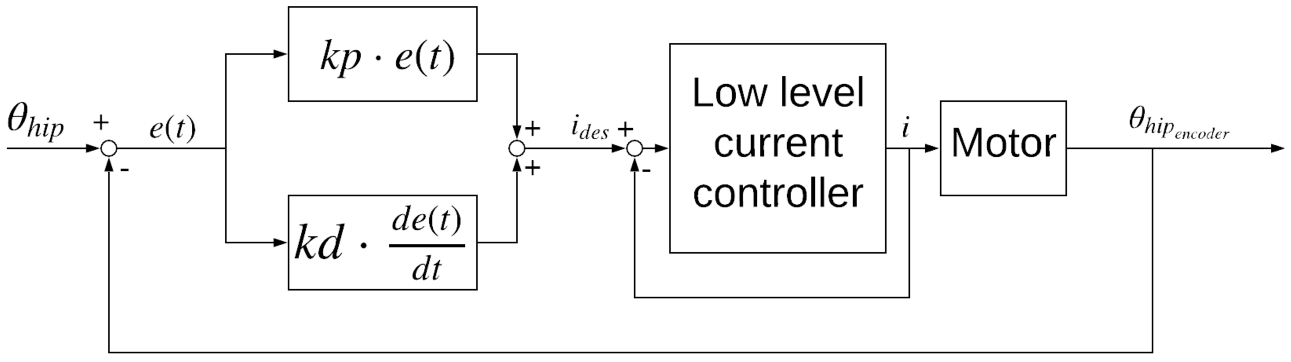

Figure S3. Controller diagram for sine wave controller in hopping experiment

2 VIDEO

Name	Description
droptestSnapshot.mp4	Side by side video of vertical drop test.
pantoSnapshot.mp4	One step hopping pantograph leg.
biartSnapshot.mp4	One step hopping biarticular leg.

Table S2. Supplementory video description.
